# Optimization through a Box–Behnken Experimental Design of the Microwave-Assisted Extraction of the Psychoactive Compounds in Hallucinogenic Fungi (*Psylocibe cubensis*)

**DOI:** 10.3390/jof8060598

**Published:** 2022-06-02

**Authors:** Curro Polo-Castellano, José Á. Álvarez, Miguel Palma, Gerardo F. Barbero, Jesús Ayuso, Marta Ferreiro-González

**Affiliations:** 1 Department of Analytical Chemistry, Faculty of Sciences, Agrifood Campus of International Excellence (ceiA3), IVAGRO, University of Cadiz, 11510 Puerto Real, Spain; curro.polocastellano@alum.uca.es (C.P.-C.); miguel.palma@uca.es (M.P.); 2 Department of Physical Chemistry, Faculty of Sciences, INBIO, University of Cadiz, 11510 Puerto Real, Spain; joseangel.alvarez@uca.es (J.Á.Á.); jesus.ayuso@uca.es (J.A.); marta.ferreiro@uca.es (M.F.-G.)

**Keywords:** hallucinogenic fungi, *Psylocibe cubensis*, psilocybin, psilocin, alkaloids, microwave-assisted extraction, Box–Behnken design

## Abstract

Hallucinogenic fungi, mainly those from the *Psilocybe* genus, are being increasingly consumed even though there is no control on their culture conditions. Due to the therapeutic potential as antidepressants and anxiolytics of the alkaloids that they produce (psilocin and psilocybin), some form of control on their production would be highly recommended. Prior to identifying their optimal culture condition, a methodology that allows their study is required. Microwave-assisted extraction method (MAE) is a technique that has proven its efficiency to extract different compounds from solid matrices. For this reason, this study intends to optimize a MAE method to extract the alkaloids found in *Psylocibe cubensis*. A surface-response Box–Behnken design has been employed to optimize such extraction method and significantly reduce time and other resources in the extraction process. Based on the Box–Behnken design, 50 °C temperature, 60% methanol as extraction solvent, 0.6 g:10 mL sample mass:solvent ratio and 5 min extraction time, were established as optimal conditions. These mild conditions, combined with a rapid and efficient UHPLC analysis result in a practical and economical methodology for the extraction of psilocin and psilocybin from *Psylocibe cubensis*.

## 1. Introduction

Natural drugs, such as hallucinogenic fungi are generally considered safe to be consumed by humans. However, since they are not subjected to any quality controls they may pose a threat for health. On the other hand, these substances can also be used to treat certain mental disorders such as anxiety [1,2], depression [3,4], or substance abuse [5,6,7] such as alcohol or tobacco because of their ability to alter consumers’ perception.

These fungi have been used in the past to trigger creativity [8] or inspiration in religious ceremonies because of their ability to alter the central nervous system (CNS) [5]. They can be found in nature, mostly in fungi of the genus *Psylocibe, Conocybe* or *Gymnopilus* [9]. Picking specimens from these genus can be a hazardous task, since they present close similarities with some lethal species [10]. *Psylocibe cubensis* is one of the most deeply studied among these fungi species. Two varieties of this species have particularly attracted researchers’ attention: *Ecuadorian*, which is native from Ecuadorian Andes [11] and *B+*, a variety that can grow fast and adapt to a diversity of culture conditions [12].

“Hallucinogenic” is a broad concept associated to the ability exhibited by certain substances to alter the perception of the CNS [8,13] and that encompasses a highly diverse group of compounds, such as psilocybin, psilocin, cannabinoids, morphine, or opium among others [14]. Their hallucinogenic effect on consumers may appear as synesthesia (mix of senses) or as a distorted perception of time among other possibilities.

Psylocibin (*O*-phosphoril-4-hydroxy-*N*,*N*-dimethyltryptamine) is the main psychotropic compound that can be obtained from the tryptophan found in “magic mushrooms”. Its active form, psilocin (4-hydroxy-*N*,*N*-dimethyltryptamine) is a serotoninergic agonist [15] that binds to (5-HT)_2A_ receptors causing the above-mentioned effects [16].

Owing to legal restrictions and other difficulties, no standard procedures to analyze these alkaloids have been developed until present. The metabolism and effects of these fungi on humans is therefore still to be investigated and the procedures for their extraction and analysis are still to be developed.

Although their detection in consumers is the main focus of the research works that have been conducted around this subject [17,18,19,20,21,22,23], an adequate quantification of the substances of interest in fungal samples should also be addressed. Microwave-assisted extraction (MAE) has already been successfully used to extract other alkaloids such as nicotinoid herbicides retained in fungal samples [24] or cannabinoids from *Cannabis* sp. [25]. It is, therefore, an extraction method that could be used for the extraction of these compounds and basically consists in the application of microwaves (0.3–300 GHz) [26] to fungi solid samples to promote the transference of psilocin and psilocybin from the matrix to a solvent.

In order to identify and quantify the alkaloids present in the resulting extracts, a detection method needs to be employed. High-performance liquid chromatography with fluorescence (FLR) [27], mass spectrometer (MS) [28], or photodiode array detection (PDA) have been the most widely used method for the detection of psilocybin and psilocin [23]. The last one registers the UV-Vis spectra of all the outgoing compounds under several wavelengths, so that compounds can be detected under optimal absorption conditions [29].

Consequently, this study aims at developing a simple, rapid, and efficient method for the quantification of psilocin and psilocybin in fungal samples that may contribute to control fungi trafficking, culture conditions, and fungal composition as the basis for further research on these alkaloids.

## 2. Materials and Methods

### 2.1. Biological Samples

Mushroom culture kits (*Psylocibe cubensis* var. *Ecuadorian* and *B+*) were obtained from an online supplier (Spaceseed S.L., El Escorial, Spain). For the study, mushrooms were lyophilized by means of a LYOALFA freeze dryer (Azbil Telstar Technologies, Terrassa, Spain) and ground using a ZM200 knofe mill (Retsch GmbH, Haan, Germany), down to <40 μm particles. The samples were stored in a freezer at −20 °C until further analysis.

### 2.2. Fungi Cultivation

Mushroom kits were cultivated according to the seller’s instructions [30]. Over the first stage, the culture humidity was kept above 85% and at temperature between 20 and 25 °C. When the first primordia started to appear on the surface of the kit, the culture bags were kept opened to let air ventilation. When the caps were about to open, the mushrooms were collected by pulling them from the stem, near the volva.

### 2.3. Solvents and Reagents

The solvents used for the extraction were mixtures of HPLC-grade methanol (Panreac Quimica, S.L.U., Castellar del Valles, Spain) and Milli-Q water, obtained through filtration by means of a MilliPore system (Bedford, MA, USA) with variable composition, ranging from 0–100% MeOH. Psilocin and psilocybin standard solutions (Sigma-Aldrich, St. Louis, MO, USA) were used to calculate the calibration curves. They were solved in methanol and kept at −32 °C. The mobile phase was formed by HPLC-grade acetonitrile (Panreac Quimica, S.L.U., Castellar del Valles, Spain), Milli-Q water and glacial acetic acid (Panreac Quimica, S.L.U., Castellar del Valles, Spain) to increase solvent acidity and avoid peak duplication.

### 2.4. Microwave-Assisted Extraction (MAE)

#### 2.4.1. Microwave-Assisted Extraction Equipment

For the microwave-assisted extraction, a MARS 240/50 microwave (OneTouch Technology, CEM Corporation, Matthews, NC, USA) was used. The equipment was fitted with a multi-location carousel including 8 positions for the extraction chambers. Every position included Teflon-coated magnets to shake the samples during the extraction process. Each chamber was also fitted with a temperature sensor and two magnetrons (one to induce microwave power on the sample and the other one to maintain the power constant during the extraction time).

#### 2.4.2. Microwave-Assisted Extraction Procedure

In order to optimize the extraction method, several extractions were performed according to the following procedure: the samples were weighted (0.30, 0.45 or 0.60 g approximately) in a microwave tube where 10 mL of the corresponding solvent were also added. Only extractions using the same solvent composition and the same temperature could be used for each extraction run. The tubes were tightly closed to prevent gas losses and placed into their respective positions on the multi-location carousel (a minimum of 8 tubes had to be processed at the same time to prevent temperature fluctuations inside the microwave). Afterwards, the specific extraction conditions for each experimental run were set: temperature (50–125 °C), extraction time (5–25 min), heating phase (3 min), extraction power (600 W), cooling stage (25 min), and maximum agitation.

After completing the extraction, the extracts were centrifuged (1702× *g*, 5 min) and the supernatant was recovered, made up to 10 mL with the extraction solvent and kept in vials at −20 °C until analysis.

### 2.5. Alkaloids Quantification

An ACQUITY UPLC^®^ H-Class System (Waters Corporation, Milford, MA, USA) with a quaternary elution system (Quaternary Solvent Manager) coupled to a photodiode detector (PAD eλ Detector) was used for the analysis of the resulting extracts. The equipment was controlled by means of the application EmpowerTM 3 (Waters Corporation, Milford, MA, USA). The column used for the separation was an Acquity UPLC^®^ BEH C18 column (1.7 μm, 2.1 mm × 100 mm, Waters, Milford, MA, USA). Prior to their analysis, the extracts were filtered using 0.22 μm syringe filters (Nylon Membrane Filter, FILTER-LAB, Barcelona, Spain).

Two phases were used, at a flow rate of 0.5 mL min^−1^, for the chromatographical separation of the compounds found in the extracts, both filtered through a 0.22 μm filter (Nylon Membrane Filter, FILTER-LAB, Barcelona, Spain) and degassed by means of an ultrasonic bath (Elma S300, Elma Schmidbauer GmbH, Singen, Germany). Phase A consisted of Milli-Q water containing 2% glacial acetic acid and phase B was a 2% glacial acetic acid-acetonitrile solution.

To identify the psilocin and psilocybin peaks, pure standards were used for reference, based on their UV-Vis absorption spectra at 260 nm. The gradient of the UHPLC method used in this study was: 0 min, 0% B; 3 min, 20% B; 5 min, 50% B; 7 min, 100% B and 13 min, 0% B. To correlate the peak areas and the compounds concentration with calibration curves (R^2^ > 0.99; y = 3161.1x + 983.33 for psilocybin and y = 6907.9x − 1567.6 for psilocin), alkaloid standard solutions were prepared in methanol at 1, 2, 4, 5, 10, and 20 mg L^−1^.

### 2.6. Box–Behnken Design (BBD) for MAE Optimization

A surface-response Box–Behnken experiment Design (BBD) determined by three factors (temperature, solvent composition, and sample-to-solvent ratio) and three levels (−1, lower; 0, intermediate; 1, higher) including three central points was used to reduce the number of experiments from 27 to 15. The procedure was to be performed under mild conditions [31] so that the degradation of the compounds of interest would be avoided. The design did not present axial points and, instead, the design points were adapted to a spherical setup.

Regarding temperature (X_1_), the values studied were 50, 75, and 100 °C. The percentages of methanol MeOH (X_2_) used in the solvent used were 60, 80, and 100% and the three levels of sample-to-solvent ratio (X_3_) established for the BBD were 0.30, 0.45, and 0.60 g in 10 mL solvent.

The response variable measured (Table 1) was absorbance (*Y*), calculated as the sum of the absorbance corresponding to both peaks (psilocybin and psilocin).

The data treatment generated a mathematical model that adjusts, as best as possible, to the experimental values that had been actually obtained from each set of extraction condition. Such model consists in a second-order polynomial equation, where the response variable is expressed as a lineal combination of the factors studied and the interactions between factors (Equation (1))

(1) $$Y=\beta_{0}+\overset{k}{\underset{i=1}{\sum }}(\beta_{i}X_{i}+\beta_{ii}X_{i}{}^{2})+\overset{n}{\underset{i<j}{\sum }}\overset{k}{\underset{i=1}{\sum }}\beta_{ij}X_{i}X_{j}+r,$$

In this equation, *Y* represents the response variable; *X*, the factors studied; $\beta_{0}$, the model constant; $\beta_{i}$, the coefficient of each main effect; $\beta_{ii}$, the coefficient of the quadratic factors; $\beta_{ij}$, the coefficient associated to the effect of the interactions between factor *i* and factor *j*, and finally *r* represents the residual value due to random error.

The fit quality of the model can be evaluated through the R^2^ coefficient, and its statistical significance can be measured by an analysis of variance (ANOVA). The application STATGRAPHICS XVI (Statgraphics Technologies, Inc., The Plains, VA, USA) was used for such analysis.

## 3. Results and Discussion

### 3.1. Alkaloids Identification Method

**Figure 1:**
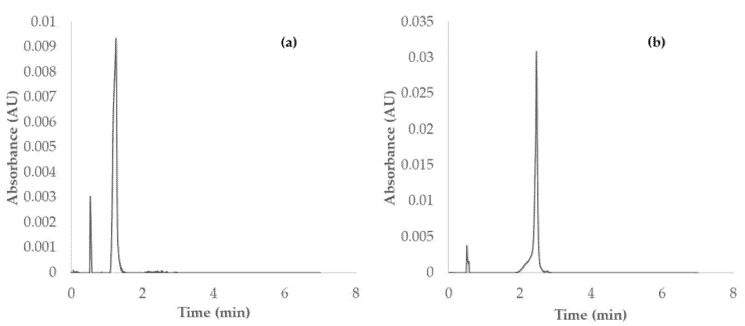
(**a**) Chromatogram of psilocybin at λ = 260 nm; (**b**) chromatogram of psilocin at λ = 260 nm.

The psychotropic compounds were identified based on their chromatograms at 260 nm (Figure 1) and the UV-Vis spectra of the pure standard solutions of psilocybin and psilocin. The retention time of psilocybin was 1.2 min, while 2.4 min was the retention time of psilocin.

### 3.2. Determination of the Range of Study for Each Factor

#### 3.2.1. Solvent Composition

This factor determines which compounds are transferred from the solid matrix to the liquid phase. Therefore, a single-factor experiment design was applied to determine the optimal water–methanol percentages (0–100%) in the solvent. All of these extractions were performed at 50 °C to minimize thermal degradation.

**Figure 2:**
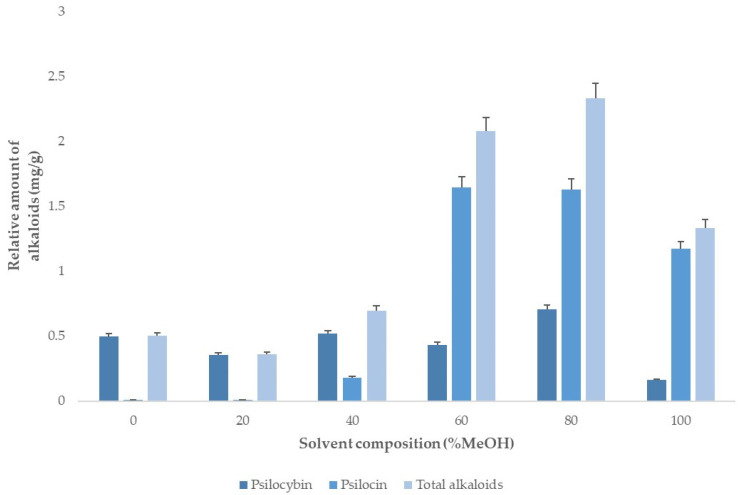
Alkaloids extraction yields obtained when using different methanol percentages in the solvent (water:methanol) (*n* = 2).

It can be observed in Figure 2 that the greatest yields were obtained when 60 and 80% methanol was added to the solvent. However, since the effects caused by possible interactions cannot be disregarded, the study range should cover from 60% to 100% methanol.

#### 3.2.2. Temperature

Psilocin and psilocybin are known to be thermolabile compounds [28], so, prior to applying the experimental design, it was necessary to verify if they remained stable within the set of extraction temperatures in the study. For that purpose, a single-factor screening experiment at temperatures going from 50 until 125 °C was performed.

The extractions were performed using a solvent containing 80% MeOH, as this solvent composition had obtained the greatest extraction yields in the previous experiment.

**Figure 3:**
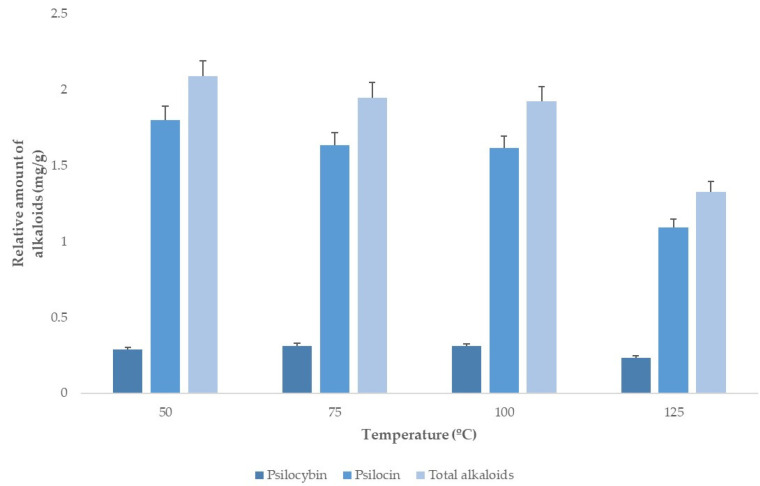
Alkaloids extraction yields at different temperatures (*n* = 2).

As Figure 3 illustrates, the maximum psilocin yield was obtained at 50 °C. It then decreased at higher temperatures, with a considerable fall at 125 °C. Since, on the other hand, psilocybin yields were very similar at all the temperatures tested, the temperature interval for the experimental design was established from 50 until 100 °C.

### 3.3. Optimization of the MAE Method

#### 3.3.1. Box–Behnken Design

The results from the statistical analysis are summarized in Table 2, from which the relevant effects can be identified, as they correspond to a *p*-value < 0.05.

**Figure 4:**
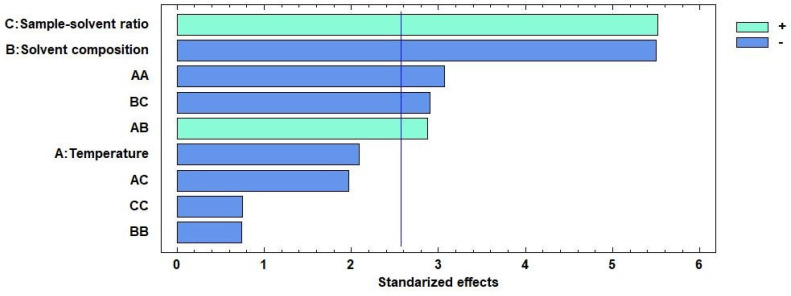
Pareto diagram of the main independent and interaction effects.

After the statistical analysis of the extractions had been completed, a Pareto diagram (Figure 4) could be constructed. The significance threshold was 2.57, so the factors that had a relevant effect on the extraction yields are sample-solvent ratio, solvent composition and the double interaction effect of temperature as well as the interactions of temperature with solvent and that of solvent with sample-solvent ratio.

The sample-solvent ratio needs to be optimized in order to obtain large yields using small solvent volumes. Nevertheless, the solvent volume must be large enough to overcome the mass transfer barrier. An optimal ratio should also allow uniform heating of the sample-solvent mixture [32].

The extraction solvent should be an alcohol-water mixture, since alcohol has a high dielectric constant that favors the absorption of microwaves. However, since pure alcohols promote to a rapid heating that may have a detrimental effect on thermolabile compounds, the amount of alcohol in the solvent should be kept at a relatively low proportion. On the other hand, the presence of water in the extraction solvent increases its permeability through the matrix barriers and decreases the dielectric constant, which slows down the heating of the solvent and, in turn, favors the stability of the alkaloids. Polarity is another factor to be taken into account, as polar substances are more soluble in polar solvents and non-polar substances dissolve better in non-polar solvents. In the case of psilocybin and psilocin, an optimal extraction solvent should present an intermediate-to-high polarity.

Finally, high temperatures contribute to increasing cellular lysis, since they induce higher pressure levels on cell walls and membranes. Furthermore, high temperatures also favor solubility and compounds transfer. However, since psilocybin and psilocin are thermolabile compounds, they are prone to suffer thermal degradation when subjected to high temperatures for a prolonged period of time. Consequently, the optimal temperature to obtain large yields should be as high as possible but, at the same time, mild enough to ensure alkaloids stability [32,33].

**Figure 5:**
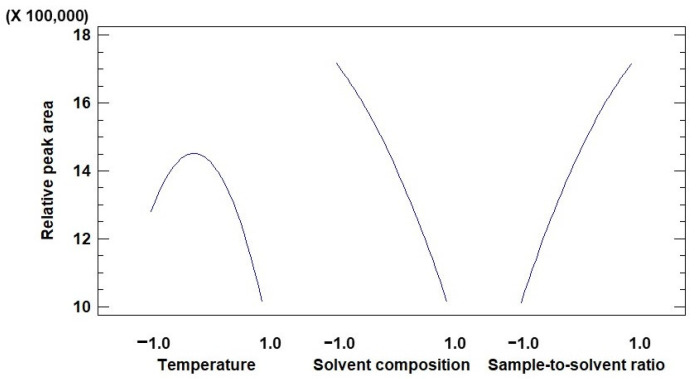
Plots representing the main effects from temperature, solvent composition and sample-solvent ratio.

A main effects plot (Figure 5) has been constructed to determine the optimal value of each factor. Thus, in the case of temperature, the optimal value should be between −1.0 and 1.0, while the optimal values for solvent composition and sample-to-solvent ratio have been established as the maximum ones in the range studied.

The R^2^ coefficient of the experimental design is 95.02% (Equation (2)), which means that there is a very small difference between the theoretical and the actual experimental values.

Y = 1.43627 × 10^6^ − 133686 × X_1_ − 351547 × X_2_ − 352690 × X_3_ − 288718 × X_1_^2^ − 260038 × X_1_X_2_ − 177962 × X_1_X_3_ − 70067.0 × X_2_^2^ + 262414 × X_2_X_3_ − 71043.0 × X_3_^2^ (2)

**Figure 6:**
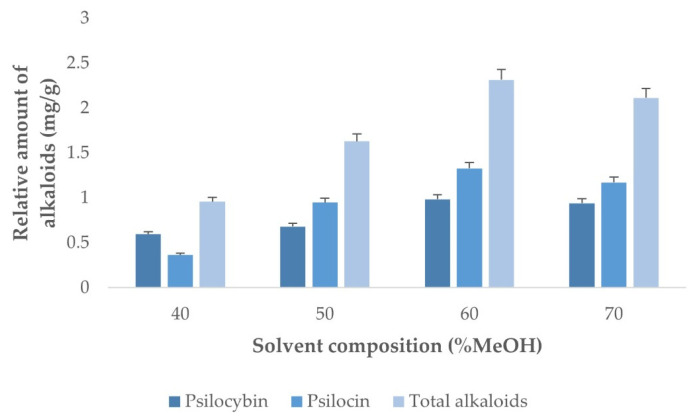
Alkaloids extraction yields obtained when using different solvent compositions (water:methanol) under the established optimal temperature and sample-to-solvent ratio values (*n* = 2).

After decoding the values obtained for temperature, solvent composition and sample-solvent ratio the optimal conditions for the MAE (Table 3) were established. The optimal values in Table 3 differ slightly from the expected ones according to Figure 6. However, such differences are explained by the fact that Figure 6 presents data that are only based on the effects of each individual factor, while for the calculation of the optimal values, the effects attributable to the different interactions have also been taken into account.

As it can be observed, the optimal temperature obtained concurs with the minimum value studied. Lower temperature levels could not be tested since the extraction equipment used cannot operate under 50 °C.

With regard to the sample-solvent ratio, the value established as optimal is the maximum one within the range studied. No higher values have been studied due to the limited amount of samples available. Given that the design was to be applied to real fungi samples and that the amount of lyophilized fungi is generally rather limited, low sample-solvent ratios had to be used for the experiment design.

On the other hand, since the solvent composition was not limited to 60% methanol in water, a new single-factor experiment had to be conducted where temperature and sample-solvent ratio would remain invariably at their optimal values, while solvent composition was tested at different methanol levels from 40% to 70% (Figure 6).

According to the data obtained there was a significant increase in extraction yields when the percentage of ethanol was increased from 50% to 60%, but the alkaloid yields decreased when the percentage of methanol went over 60%. Therefore, it was determined that 60% methanol was the optimal percentage of methanol in the solvent and, therefore, it was not necessary to perform a new BBD to optimize this factor.

#### 3.3.2. Optimal Extraction Time

**Figure 7:**
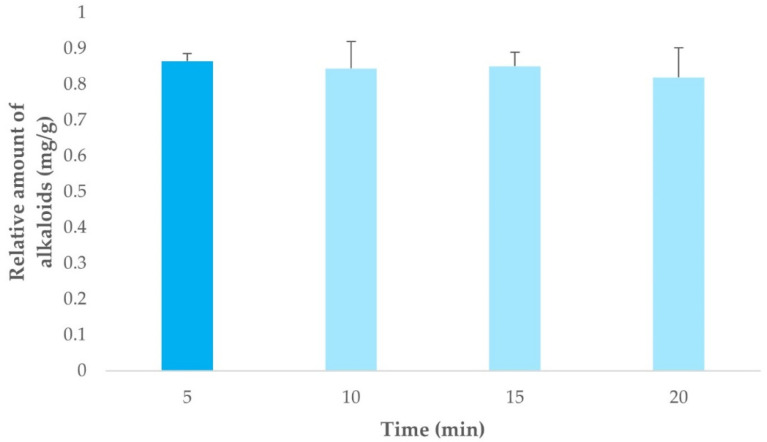
Extraction yields obtained (*n* = 3) under optimal extraction conditions while varying the extraction times.

In order to determine the optimal extraction time, a number of extractions were performed in triplicate under the optimal conditions that had been established (temperature, solvent composition, and sample-solvent ratio) using 5, 10, 15, and 20 min as extraction time. The mean value of the three extractions performed at the same temperature are displayed in Figure 7, where error bars (based on standard deviation (SD)) have been included.

Longer extraction times were not considered for the screening design as a significant thermal degradation of the alkaloids took place when longer extraction times were used, even at low temperatures [32,33]. Furthermore, longer extraction times imply a greater consumption of resources, which is not a desirable aspect in an optimized analytical method.

Since yields did not vary significantly as the extraction time was changed, the shortest time (5 min), with lesser resources demand, was selected to perform the rest of the extractions.

#### 3.3.3. Repeatability and Intermediate Precision

The precision of the extraction method was quantified in terms of repeatability and intermediate precision. Eight extractions per day were performed under optimal conditions on three consecutive days to make a total of 24 extractions. Finally, the repeatability was determined as 2.82% and the intermediate precision was calculated as 3.65%. Both coefficients are below 5%, which means that the extraction method can be considered repeatable and with a good intermediate precision, since 5% is the variation limit generally accepted [34].

### 3.4. Quantification of Alkaloids in Real Samples

**Figure 8:**
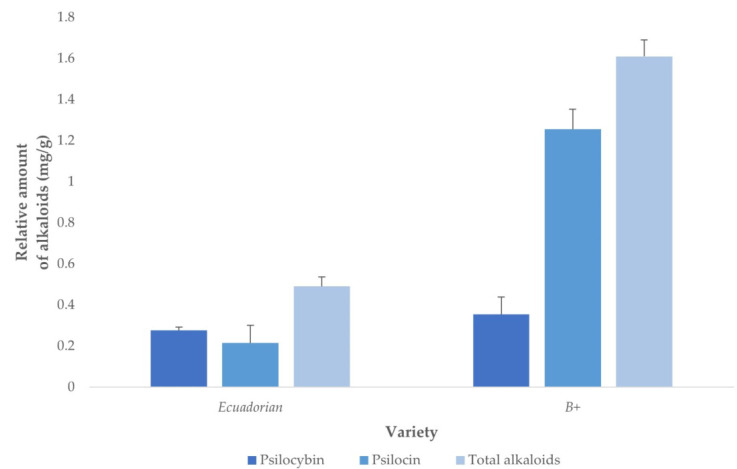
Psilocybin, psilocin and total alkaloid yields obtained through the application of the optimized MAE method to fungal samples of *Ecuadorian* and *B+* varieties (*n* = 8).

The optimized method was applied to fungal samples of *P. cubensis* var. *Ecuadorian* and var. *B+* (Figure 8) for comparison purposes. *B+* showed a greater potential for alkaloid production (1.609 ± 0.081 mg/g) than *Ecuadorian* (0.490 ± 0.047 mg/g), probably due to its ability to get adapted to variable culture conditions, such as temperature, humidity, and light within the recommended ranges.

Although ultrasound-assisted extraction (UAE) has, so far, been the most frequently used method to extract psilocybin and psilocin from dried mushroom samples, other more efficient methods are still to be investigated. In this regard, MAE achieved in our study a maximum yield of 1.609 ± 0.081 mg/g relative alkaloids (1.255 ± 0.098 mg/g psilocin and 0.354 ± 0.085 mg/g psilocybin). This extraction yield is greater than the mean yields reported by previous studies where UAE was the extraction method employed; Gotvaldová et al. obtained 0.54 mg/g psilocybin and 0.25 mg/g psilocin by vortexing dried mushroom samples [28], Laussmann et al. reported 1.151 ± 0.228 mg/g psilocybin and 0.126 ± 0.066 mg/g psilocin by UAE [35] and Tsujikawa et al. reported even lower yields of 0.835 ± 0.465 mg/g psilocybin and 0.280 ± 0.140 mg/g psilocin [36] when two UAE cycles were employed.

The main difference between the previous results and the results obtained from this study is that the previous works reported greater psilocybin yields than psilocin ones. However, in the present study, the trend seems to be the opposite, which might be explained by the thermal instability of psilocybin, which becomes rapidly dephosphorylated into psilocin when subjected to a high temperature for a long time.

All things considered, MAE has proven to be a comparable method to UAE for the efficient extraction of alkaloids from dry mushroom samples, since it uses short times and low microwave power to obtain high extraction yields

## 4. Conclusions

Microwave-assisted extraction is an appropriate method to extract alkaloids from fungi under mild conditions. A low energy consumption, because of the low extraction temperature used and the employment of a solvent with a high water content make this a rather environmentally friendly extraction method. The sample-solvent ratio is also low, which makes this extraction method suitable for analysis where the amount of sample available is a limiting factor.

MAE is also a rapid (5 min of processing time per sample) and effective method, that can produce large extraction yields, comparable to those obtained by UAE. This method can also be standardized, as it shows a high repeatability and intermediate precision.

## Figures and Tables

**Table 1:** Experimental and predicted values for absorbance based on the Box–Behnken design for microwave-assisted extraction.

Run	Factors			Response (Sum of Both Peaks)	
	X_1_	X_2_	X_3_	Predicted	Experimental
1	0	1	−1	1,313,823	894,261
2	−1	0	−1	1,487,617	535,292
3	1	−1	0	1,317,156	931,988
4	1	0	−1	1,399,065	887,638
5	1	0	1	2,460,369	1,261,792
6	0	−1	1	1,316,109	2,220,876
7	0	0	0	1,436,270	1,540,337
8	−1	−1	0	1,569,860	1,983,228
9	1	1	0	2,540,326	691,809
10	0	0	0	1,436,270	1,370,605
11	0	1	1	2,544,031	1,050,115
12	−1	1	0	1,752,878	702,897
13	−1	0	1	1,837,073	1,621,296
14	0	−1	−1	1,135,557	1,015,368
15	0	0	0	1,436,270	1,397,853

**Table 2:** Analysis of variance of the polynomic model adjusted to the extraction yield.

Factor	Factor Code	Coefficients	Sum of Squares (10^11^)	Degrees of Freedom	Mean Square (10^10^)	*F*-Value	*p*-Value
Model		1.43627 × 10^6^					
A: Temperature	X_1_	−133,686	1.42975	1	14.2975	4.37	0.0908
B: Solvent composition	X_2_	−351,547	9.88684	1	98.8684	30.23	0.0027
C: Sample-solvent ratio	X_3_	352,690	9.95122	1	99.5122	30.42	0.0027
AA	X_1_^2^	−288,717	3.07783	1	30.7783	9.41	0.0279
AB	X_1_ X_2_	260,038	2.70479	1	27.0479	8.27	0.0348
AC	X_1_ X_3_	−177,962	1.26683	1	12.6683	3.87	0.1062
BB	X_2_^2^	−70,067.0	0.18127	1	1.8127	0.55	0.4901
BC	X_2_ X_3_	−262,414	2.75443	1	27.5443	8.42	0.0337
CC	X_3_^2^	−71,043.0	0.186355	1	1.86355	0.57	0.4844
Lack of fit			1.46928	3	4.89759	5.89	0.1485
Pure error			0.166177	2	0.830885		
Total correlation			32.8692	14			

**Table 3:** Optimal extraction conditions.

Factor	Optimal Value
Temperature (°C)	50
Sample-solvent ratio (g/10 mL)	0.60
Solvent composition (%MeOH)	60

## Data Availability

The data presented in this study is contained within the article.
